# Ketogenic diet as a therapeutic strategy for neurodegenerative diseases: from mechanisms to translational challenges

**DOI:** 10.1186/s40035-026-00557-1

**Published:** 2026-05-25

**Authors:** Ana Margarida Salgueiro, Marisa Ferreira-Marques, Rodrigo F. N. Ribeiro, Sara M. Lopes, Dina Pereira, Daniela G. Costa, Magda M. Santana, Luís Pereira de Almeida, Cláudia Cavadas

**Affiliations:** 1 Center for Neuroscience and Cell Biology (CNC-UC), University of Coimbra, 3004-504 Coimbra, Portugal; 2 Center for Innovation in Biomedicine and Biotechnology (CIBB), University of Coimbra, 3004-504 Coimbra, Portugal; 3 Faculty of Pharmacy, University of Coimbra (FFUC), 3000-548 Coimbra, Portugal; 4 Gene Therapy Center of Excellence (GeneT), 3004-504 Coimbra, Portugal

**Keywords:** Ketogenic diet, Neurodegenerative diseases, Neuroinflammation, Therapeutic strategy

## Abstract

The ketogenic diet (KD) is increasingly recognized as a promising therapeutic strategy for neurodegenerative disorders because of its multifaceted impacts on key pathophysiological mechanisms. This review explores the molecular pathways through which KD may protect against neurodegeneration, including the use of ketone bodies as alternative energy substrates, reduction of oxidative stress and inflammation, modulation of autophagy and protein aggregation, and impact on the gut microbiome. The potential benefits of KD are explored across neurodegenerative diseases such as Alzheimer’s disease, Parkinson’s disease, Huntington’s disease, amyotrophic lateral sclerosis, and multiple sclerosis, based on both preclinical and clinical evidence that supports its feasibility. However, challenges in long-term safety, patient adherence, and clinical practicality limit its widespread adoption. This review underscores the potential of KD for treating neurodegeneration on the basis of current scientific evidence while highlighting the need for further research to optimize its application and address existing gaps.

## Introduction

Neurodegenerative diseases are chronic, progressive disorders characterized by gradual degeneration of neurons in the central nervous system (CNS). These include conditions such as Alzheimer's disease (AD), Parkinson's disease (PD), Huntington's disease (HD), amyotrophic lateral sclerosis (ALS), and multiple sclerosis (MS) [1]. Despite presenting different clinical features, these diseases share common pathological mechanisms. A critical feature is neuronal death, which results from the loss of neurons' ability to maintain their function and viability. Mitochondrial dysfunction plays a central role, leading to impaired oxidative phosphorylation, reduced adenosine triphosphate (ATP) generation, and increased oxidative stress, all contributing to cellular damage and death [2]. Additionally, deficits in energy metabolism, such as impaired glucose utilization, further exacerbate neuronal vulnerability [3]. Neuroinflammation is also a key player, characterized by microglial activation and production of pro-inflammatory cytokines, which exacerbate neuronal dysfunction and degeneration [4]. In addition to microglial activation, astrocytic dysfunction contributes to synaptic and metabolic impairment in neurodegenerative disorders. Astrocytes regulate glutamate clearance and metabolic support to neurons, and disruption of these functions promotes excitotoxic stress and increases neuronal vulnerability [5].

Impaired synaptic plasticity [6], autophagy dysfunction [7], and accumulation of misfolded proteins such as β-amyloid (Aβ), α-synuclein and mutant huntingtin [8] are other shared features across diseases. Furthermore, epigenetic dysregulation has been described in neurodegenerative models, including alterations in chromatin structure and histone acetylation that influence gene expression linked to metabolic stress responses and neuronal survival [9]. In addition, growing evidence suggests that the gut-brain axis disruption, such as changes in microbiota composition and increased intestinal permeability, can contribute to neuroinflammation [10, 11].

**Fig. 1:**
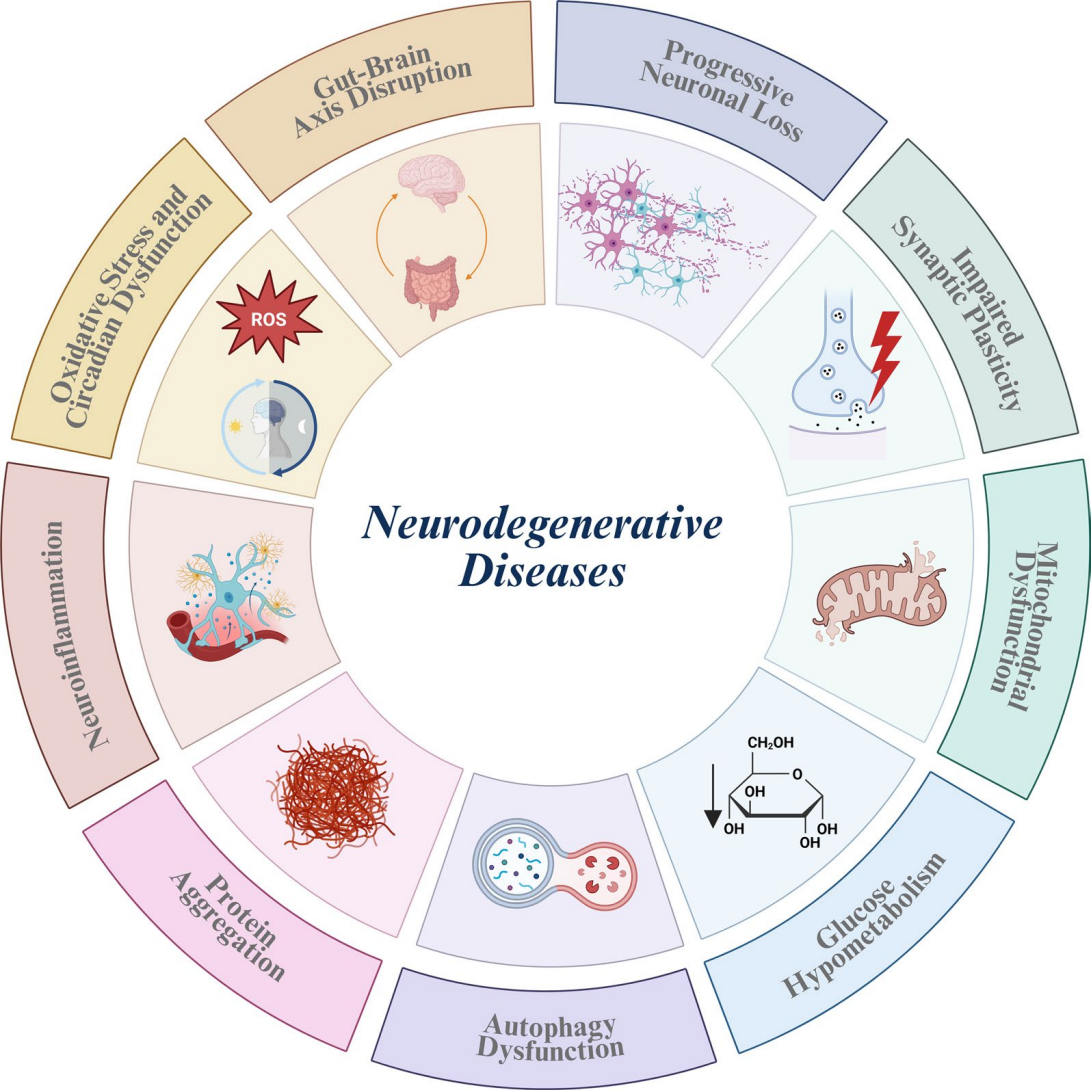
Key pathophysiological mechanisms underlying neurodegenerative diseases, including Alzheimer’s disease, Parkinson’s disease, Huntington’s disease, amyotrophic lateral sclerosis and multiple sclerosis. Illustration was created using BioRender.com

Circadian rhythm alterations have been identified as a common hallmark in neurodegenerative disorders [12, 13]. Experimental evidence indicates that disruption of core clock gene expression affects mitochondrial function, redox balance, and inflammatory signaling, thereby linking circadian dysregulation to metabolic impairment and neuronal vulnerability [14, 15]. Importantly, converging clinical and preclinical data support a bidirectional interplay whereby circadian disruption and sleep disorders not only emerge early in the disease course, but also aggravate neurodegenerative processes and deeply affect the quality of life of patients and caregivers, underscoring the potential of circadian-based interventions as disease-modifying strategies [13]. Together, these mechanisms drive the progressive motor, cognitive, and functional decline observed in affected individuals (Fig. 1).

While current treatments may alleviate specific physical or cognitive symptoms, they fail to halt disease progression, and curative solutions remain elusive. This highlights the urgent need for innovative therapeutic strategies to address these debilitating disorders. Recently, the ketogenic diet (KD) has emerged as a promising metabolic intervention with potential preventive and therapeutic applications in neurological disorders [16, 17]. By modulating mitochondrial function, oxidative stress, and neuroinflammation, KD may exert preventive effects by enhancing metabolic resilience during earlier phases of disease progression and contribute to symptom management in established neurodegenerative conditions [18–20]. Therefore, KD may represent a valuable adjunctive metabolic strategy, complementing disease-specific pharmacological therapies by targeting shared metabolic and inflammatory pathways across neurodegenerative disorders [21, 22].

This review aims to evaluate KD as a therapeutic intervention for neurodegenerative diseases by examining its effects on pathological mechanisms, such as mitochondrial dysfunction, neuroinflammation, and alterations in energy metabolism, to assess its feasibility and potential in managing neurological disorders.

## Mechanisms of action of KD in neurodegeneration

The classical KD is a high-fat, low-carbohydrate, and adequate-protein dietary regimen designed to mimic the metabolic effects of fasting without significant calorie restriction, thereby promoting an anabolic state [23]. In a typical KD, approximately 80% of the total energy intake comes from fat, 15% from protein, and only 5% from carbohydrates. These proportions may vary depending on the specific KD protocol employed, with common fat-to-carbohydrate/protein ratios being 3:1 or 4:1. Higher ratios, although more restrictive, are generally more effective [24–26].

**Fig. 2:**
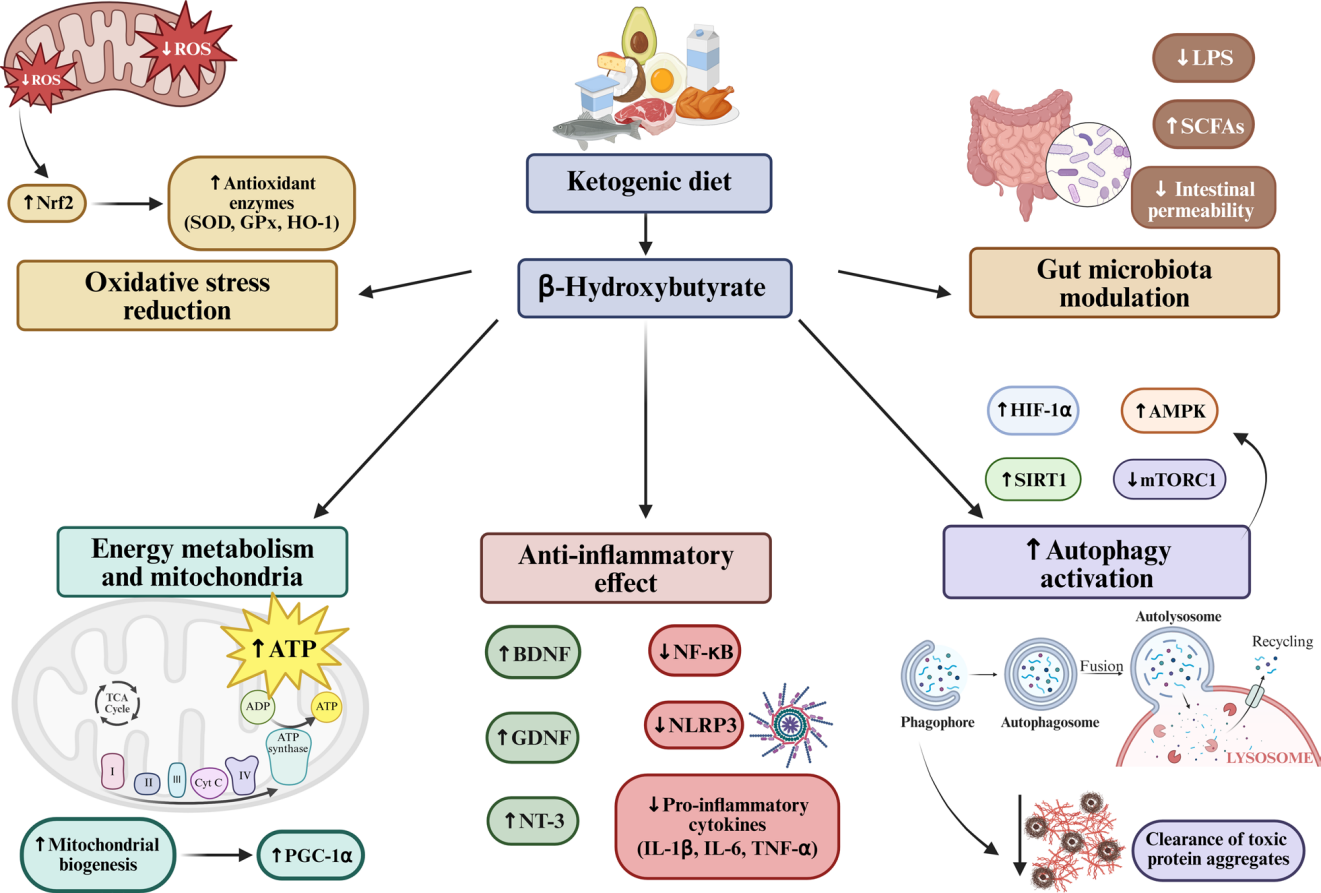
Neuroprotective mechanisms of ketogenic diet-derived β-hydroxybutyrate (BHB) in neurodegenerative diseases. BHB is a major circulating ketone body produced from ketogenic diet (KD) and plays a central role in mediating its neuroprotective properties. BHB reduces oxidative stress by suppressing mitochondrial reactive oxygen species (ROS) and activating the nuclear factor erythroid 2-related factor 2 (Nrf2) pathway, which increases the production of antioxidant enzymes such as superoxide dismutase (SOD), glutathione peroxidase (GPx), and heme oxygenase-1 (HO-1). BHB also enhances mitochondrial function and energy metabolism by increasing adenosine triphosphate (ATP) production and driving mitochondrial biogenesis via the expression of peroxisome proliferator-activated receptor γ coactivator 1-α (PGC-1α). BHB mitigates neuroinflammation through increasing the release of neuroprotective factors such as brain-derived neurotrophic factor (BDNF), glial cell line-derived neurotrophic factor (GDNF), and neurotrophin-3 (NT-3), inhibiting the nuclear factor kappa B (NF-κB) pathway and the nucleotide-binding oligomerization domain-, leucine-rich repeat- and pyrin domain-containing protein 3 (NLRP3) inflammasome activation, and decreasing pro-inflammatory cytokines such as interleukin-1 beta (IL-1β), interleukin-6 (IL-6), and tumor necrosis factor alpha (TNF-α). Moreover, BHB regulates key pathways involved in autophagy and protein clearance, such as hypoxia-induced factor 1α (HIF-1α), Sirtuin-1 (SIRT1), AMP-activated protein kinase (AMPK), and mammalian target of rapamycin complex 1 (mTORC1), facilitating the removal of toxic protein aggregates like β-amyloid and tau. Finally, BHB modulates the gut microbiota by decreasing lipopolysaccharides (LPS) and increasing short-chain fatty acids (SCFAs), strengthening the gut-brain axis. Illustration created using BioRender.com

The most commonly used form of KD includes long-chain fatty acids and was initially introduced by Wilder in 1921 for the treatment of drug-resistant epilepsy in children [27]. The high-fat composition of the diet induces a metabolic state known as “nutritional ketosis”, wherein the body shifts its primary energy source from glucose to ketone bodies, such as β-hydroxybutyrate (BHB). This shift influences several pathways implicated in neurodegenerative diseases, including energy metabolism, oxidative stress, neuroinflammation, autophagy, and gut microbiota composition (Fig. 2) [23]. Importantly, nutritional ketosis is characterized not only by elevated circulating ketone bodies, but also by reduced blood glucose levels which may independently influence neuronal metabolism and cellular stress responses. High glycolytic flux increases mitochondrial nicotinamide adenine dinucleotide hydrogen (NADH) production and electron transport chain activity, which can enhance reactive oxygen species (ROS) generation [28]. Therefore, reduced glucose utilization may decrease mitochondrial ROS production and attenuate oxidative stress. Dysregulation of glucose metabolism promotes protein glycation and the formation of advanced glycation end-products, which exacerbate the Aβ-induced neuronal toxicity and oxidative stress in experimental models [29, 30]. Therefore, by reducing systemic glucose availability, KD may theoretically limit glycation reactions and attenuate related neurotoxic mechanisms. Decreased glucose levels may also mitigate the dysregulation of insulin signaling implicated in AD [31]. Collectively, these findings suggest that reduced glucose metabolism may contribute, alongside ketone body signaling, to the potential neuroprotective effects of KD in certain disease contexts.

In addition to ketone bodies and glucose modulation, KD may also influence the metabolism of branched-chain amino acids (BCAAs). BCAAs, including leucine, isoleucine, and valine, are essential amino acids involved in energy metabolism and nutrient-sensing pathways [32]. Alterations in circulating and central BCAA levels have been reported in neurodegenerative disorders. Experimental evidence from AD models suggests that excessive BCAA availability may aggravate neuropathology. In transgenic AD mice, BCAA supplementation combined with a high-fat diet increased mortality and exacerbated tau phosphorylation, whereas reduction of dietary BCAA improved cognitive performance [33, 34]. In contrast, clinical metabolomic analyses of KD have reported increases in specific BCAAs, such as valine, and improvement of AD-related risk factors such as increased HDL-C and reduced BMI, suggesting that BCAA dynamics may differ depending on the disease stage and metabolic context [35]. These observations indicate that alterations in amino acid metabolism may contribute to the broader metabolic milieu induced by KD, although their precise impact appears to vary across experimental and clinical settings.

### Ketone bodies: an alternative energy source

Ketone bodies, which are produced during KD or fasting, serve as an alternative energy source for the brain, particularly in conditions where glucose metabolism is impaired, such as in neurodegenerative diseases. Ketone bodies, including acetoacetate (AcAc), BHB, and acetone, are synthesized in the liver from the oxidation of fatty acids when glucose availability is limited, through the process of ketogenesis [36]. Although ketone bodies are often discussed collectively, their biological effects are not identical. BHB, the predominant ketone body in circulation, not only serves as an energy substrate, but also inhibits class I histone deacetylases, suppresses NLRP3 (nucleotide-binding oligomerization domain-, leucine-rich repeat- and pyrin domain-containing protein 3) inflammasome activation, and modulates nuclear factor kappa B (NF-κB) activity, thereby influencing gene expression related to antioxidant defence, inflammation, and mitochondrial function [21, 37, 38]. In contrast, AcAc, the main ketone body produced in the liver, primarily acts as a metabolic substrate, being directly converted to acetyl coenzyme A (acetyl-CoA) to fuel the tricarboxylic acid (TCA) cycle. It also contributes to the cellular redox balance through its interconversion with BHB. Acetone, a spontaneous decarboxylation product of AcAc, appears to have limited direct signaling activity and is generally considered a minor metabolic by-product [36]. Experimental evidence suggests that ketone bodies exert both bioenergetic effects, such as enhancing mitochondrial ATP production [39, 40], and signaling effects mediated primarily by BHB [21, 37]. The relative importance of these mechanisms may differ across disease models.

**Fig. 3:**
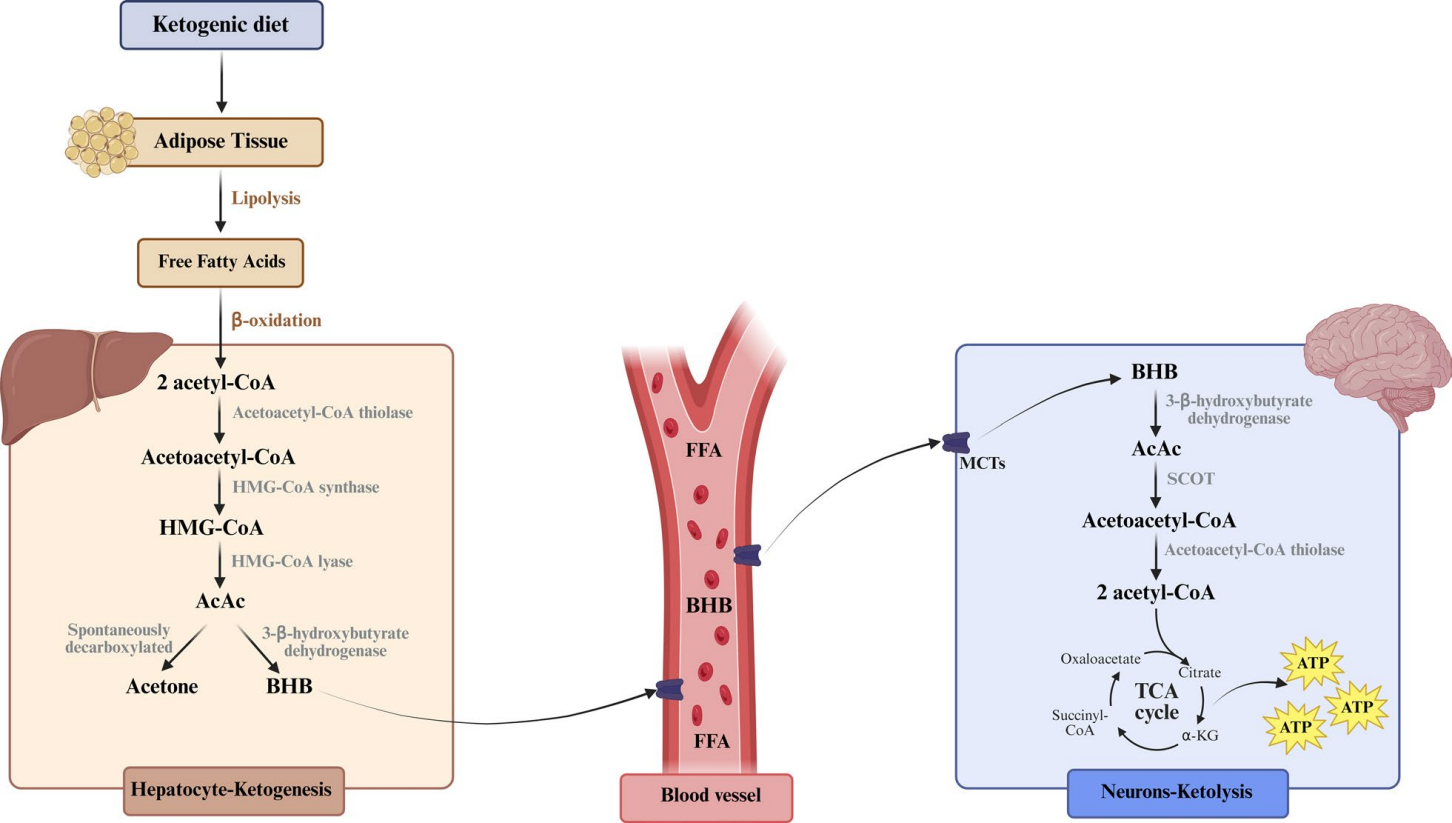
Biochemistry of ketone body synthesis and utilization during ketogenic diet and fasting. Carbohydrate restriction promotes adipose tissue lipolysis, releasing free fatty acids (FFA) that undergo β-oxidation in hepatic mitochondria, generating acetyl coenzyme A (acetyl-CoA). Excess acetyl-CoA is converted to acetoacetyl-CoA, then to 3-hydroxy-3-methylglutaryl-CoA (HMG-CoA) via mitochondrial HMG-CoA synthase, and subsequently to acetoacetate (AcAc) by HMG-CoA lyase. AcAc is either reduced to β-hydroxybutyrate (BHB) by 3-β-hydroxybutyrate dehydrogenase or spontaneously decarboxylated to acetone. Ketone bodies are released into circulation and transported into extrahepatic tissues, including the brain, through monocarboxylate transporters (MCTs). In target tissues, BHB and AcAc are reconverted to acetyl-CoA via succinyl-CoA:3-ketoacid CoA transferase (SCOT), entering the tricarboxylic acid (TCA) cycle to support mitochondrial oxidative phosphorylation and adenosine triphosphate (ATP) production. Created with BioRender.com. Adapted from [42]

During the initial stages of KD and fasting, energy relies primarily on glucose metabolism. As hepatic glycogen stores become depleted, adipose tissue releases fatty acids, which undergo β-oxidation in the liver to produce acetyl-CoA. The acetyl-CoA typically enters the TCA cycle for ATP production. However, as glucose availability declines, oxaloacetate is increasingly used for gluconeogenesis to maintain blood glucose levels. This reduces its availability for the TCA cycle, limiting acetyl-CoA entry, which leads to its accumulation. The excess acetyl-CoA is consequently redirected toward the synthesis of ketone bodies in hepatocytes [36] through sequential enzymatic reactions involving acetoacetyl-CoA thiolase, mitochondrial 3-hydroxy-3-methylglutaryl-CoA (HMG-CoA) synthase, and HMG-CoA lyase [41]. The first ketone body to be produced is AcAc, which can spontaneously be converted to acetone or be enzymatically converted to BHB via 3-β-hydroxybutyrate dehydrogenase [42]. Ketone bodies are transported into the bloodstream by monocarboxylate transporters and are used by the brain, heart, and muscle as an alternative energy source [23]. Upon cellular uptake, ketone bodies are metabolized back to acetyl-CoA, which enters the TCA cycle to support ATP production via mitochondrial oxidative phosphorylation [36, 41] (Fig. 3).

The brain's transition from glucose oxidation to ketone body utilization requires an adaptation period, after which ketone bodies can supply approximately 60%–70% of the brain's energy demand, supporting cognitive and functional performance [43]. Under normal conditions, the blood ketone body levels are typically below 0.3 mmol/L but can rise to 7–8 mmol/L during prolonged fasting or nutritional ketosis, providing a stable energy source for the CNS without affecting the blood pH, which remains at 7.4. Ketone bodies are efficiently utilized in the CNS, preventing their concentration from exceeding 8 mmol/L, and maintaining the metabolic balance [44].

Metabolism of ketone bodies produces acetyl-CoA, which directly enters the TCA cycle, bypassing glycolysis and pyruvate dehydrogenase. This may support efficient ATP production when glucose metabolism is impaired [3, 39]. In addition to their role in energy production, ketone bodies such as BHB act as signaling molecules through multiple mechanisms. As an endogenous ligand of hydroxycarboxylic acid receptor 2 (HCA2), a G-protein-coupled receptor expressed on immune cells, BHB has been shown to modulate inflammatory responses in an experimental model of neuroinflammation [45]. Beyond receptor-mediated signaling, BHB mediates post-translational modifications by, e.g., inhibiting class I histone deacetylases, leading to increased histone acetylation and transcriptional activation of antioxidant and mitochondrial regulators such as peroxisome proliferator-activated receptor γ coactivator 1-α (PGC-1α) [37, 46]. In addition, BHB induces lysine β-hydroxybutyrylation, a histone modification that regulates genes involved in redox homeostasis and energy metabolism [47]. Emerging evidence further suggests that β-hydroxybutyrylation of TCA cycle enzymes, including citrate synthase and succinyl-CoA ligase, may enhance enzymatic activity and increase mitochondrial ATP production in an animal model of AD, thereby linking post-translational modifications to bioenergetic regulation [48]. Collectively, these mechanisms highlight the dual role of BHB as both an energy substrate and a modulator of cellular metabolic and stress-response pathways.

### Reduction of oxidative stress and modulation of circadian rhythm

Oxidative stress, characterized by excessive generation of ROS, reduced ATP production, and mitochondrial dysfunction, is a key feature of neurodegenerative diseases. KD alleviates oxidative stress through multiple mechanisms, including the enhancement of mitochondrial biogenesis and increased ATP production [20, 39].

Ketone bodies, such as BHB and AcAc, exhibit neuroprotective effects by reducing ROS production and improving mitochondrial stability. This is achieved through the promotion of NADH oxidation and inhibition of mitochondrial permeability transition, both of which are crucial factors for maintaining cellular energy and survival [19].

In addition, KD enhances translocation of nuclear factor erythroid 2-related factor 2 (Nrf2) and increases expression of glutathione peroxidase and heme-oxygenase-1 in an AD mouse model, suggesting activation of Nrf2-dependent antioxidant responses in this experimental context. These alterations are associated with reduced oxidative stress in the brain [22]. Furthermore, ketone bodies enhance the activity of mitochondrial uncoupling protein 2, reducing electron leakage and ROS formation, finally contributing to improved mitochondrial function [49].

Ketone body BHB activates sirtuin-1 (SIRT1), a key metabolic sensor that mitigates oxidative stress and reduces neuronal apoptosis [50]. This effect is partly mediated through upregulation of anti-apoptotic proteins such as B-cell lymphoma 2 (Bcl-2), and downregulation of pro-apoptotic proteins such as tumour protein 53 and Bcl-2-associated X protein, collectively enhancing neuronal survival [50]. Additionally, SIRT1 modulates the activity of forkhead box O (FOXO) transcription factors, which activate the expression of enzymes involved in ROS detoxification, including SOD2 (superoxide dismutase 2) and catalase, thereby attenuating oxidative stress-induced apoptosis [51].

Beyond its role in oxidative stress, SIRT1 also regulates the circadian clock, a system increasingly recognized as vulnerable to dysfunction in neurodegenerative disorders [52]. Circadian rhythms are endogenous, approximately 24-h oscillations that govern key physiological and metabolic processes in synchrony with environmental cues. Disruptions in circadian rhythms are frequently observed across various neurodegenerative conditions, and mainly involve the suprachiasmatic nucleus, a central brain structure responsible for coordinating biological rhythms, as recently demonstrated by our group in spinocerebellar ataxia type 3 [12]. Notably, circadian impairments contribute to systemic dysregulation and may exacerbate disease progression [52].

Experimental studies have shown that dietary supplementation with ketone esters or low-percentage fat enrichment can restore locomotor rhythmicity, normalize expression of SIRT1 and core clock genes, and ameliorate neurodegeneration [53]. Although this intervention relied on ketone supplementation, rather than a classical ketogenic diet, these findings suggest that KD may promote circadian homeostasis to treat circadian rhythm disturbances in neurodegenerative diseases.

### Anti-inflammatory effects

Neuroinflammation plays a significant role in the progression of neurodegenerative diseases. KD has demonstrated anti-inflammatory effects. A primary mechanism is the inhibition of the NLRP3 inflammasome by BHB. The NLRP3 inflammasomes are central to the initiation of inflammatory responses by activating caspase-1 and promoting the release of pro-inflammatory cytokines, particularly interleukin (IL)-1β and IL-18. Experimental studies have shown that BHB suppresses NLRP3 inflammasome activation in response to defined inflammatory stimuli (urate crystals, ATP and lipotoxic fatty acids), suggesting that this effect depends on the cellular activation state and the inflammatory context [21]. By suppressing NLRP3 activity, KD reduces cytokine production and attenuates neuroinflammatory responses in animal models of AD and PD [54, 55]. In addition, BHB acts as an endogenous ligand of HCA2. In a mouse model of cerebral ischemia, HCA2 activation by BHB promotes a neuroprotective phenotype of macrophages to produce prostaglandin D2, which exerts neuroprotective effects, suggesting a potential immunomodulatory mechanism that may be relevant in inflammatory contexts [45]. BHB also suppresses the NF-κB pathway, reducing inflammatory responses and creating a neuroprotective environment. This emphasizes the potential therapeutic benefits of KD in neurodegenerative diseases [38].

KD decreases the expression of pro-inflammatory proteins, such as cyclooxygenase-2 (COX-2) and inducible nitric oxide synthase, which modulate the activity of cytokines IL-1β, IL-2, IL-4, IL-6 and TNF-α [38, 56]. Additionally, KD activates peroxisome proliferator-activated receptor γ, which suppresses NF-κB expression and mitigates neuronal damage associated with N-methyl-D-aspartate (NMDA) excitotoxicity [57, 58].

KD has been reported to increase the levels of neurotrophic factors, particularly brain-derived neurotrophic factor (BDNF), which plays a critical role in neuronal growth, survival, and synaptic plasticity. Mechanistically, BHB enhances BDNF expression in the hippocampus of mice through epigenetic modulation, including inhibition of class I histone deacetylases, and through activation of CREB (cyclic adenosine monophosphate (cAMP) response element-binding protein)-dependent transcriptional pathways [59]. In contrast, although increases in other neurotrophic factors such as neurotrophin-3 and glial cell line-derived neurotrophic factor have been reported in certain experimental settings, the precise molecular mechanisms underlying their regulation remain less clearly defined [60].

### Autophagy enhancement and reduction of protein aggregation

The accumulation of misfolded proteins, such as Aβ and α-synuclein, is a hallmark of several neurodegenerative disorders, such as AD and PD. Autophagy is a cellular process responsible for degrading and recycling cellular components, but this process is often impaired in neurodegenerative disorders, leading to failure in clearance of toxic protein aggregates [7].

Studies have shown that KD enhances autophagy in the brain through activation of SIRT1, HIF-1α (hypoxia-induced factor 1α) and AMP-activated protein kinase (AMPK), and inhibition of the mammalian target of rapamycin complex 1 (mTORC1). These actions facilitate the clearance of toxic protein aggregates, reduce cellular stress, and may contribute to slowing disease progression in animal models of neurodegeneration. Mechanistically, SIRT1 may promote autophagy directly through its deacetylase activity on autophagy-related proteins, and indirectly through modulating upstream nutrient-sensing pathways such as AMPK–mTORC1, depending on the cellular energy status and the availability of nicotinamide adenine dinucleotide^+^ [7, 61, 62]. Moreover, SIRT1 enhances the expression of PGC-1α, thereby promoting mitochondrial biogenesis under conditions of energetic stress [63]. SIRT1 has also been shown to facilitate tau deacetylation and clearance in transgenic models of AD [64].

Moreover, KD enhances the activity of molecular chaperones and proteins critical for the maintenance of cellular protein homeostasis. These chaperones prevent protein misfolding and aggregation, aid in the proper transport of proteins across cellular membranes, and target damaged proteins for degradation. They also regulate anti-apoptotic and anti-inflammatory factors, thereby contributing to neuronal health and resilience [8, 65].

### Gut microbiota modulation

The gut–brain axis plays a pivotal role in the progression of neurodegenerative diseases. Disruption of this axis, often due to gut microbiome dysbiosis, leads to intestinal dysfunction and systemic inflammation. Alterations in gut microbiota composition have been consistently reported in patients with neurodegenerative disorders, and are characterized by reduced levels of neuroprotective compounds, including neurotransmitters and short-chain fatty acids (SCFAs), alongside elevated levels of pro-inflammatory cytokines and harmful compounds such as lipopolysaccharides (LPS). These changes exacerbate neuronal damage and contribute to the progression of neurodegenerative conditions [10, 11, 66]. Importantly, SCFAs such as acetate, propionate, and butyrate, are central mediators of gut–brain communication, regulating immune responses and maintaining intestinal barrier integrity [66, 67].

Emerging evidence indicates that the KD modulates the composition of gut microbiota. Increased circulating plasma ketone bodies lead to the inhibition of *Bifidobacterium* growth and decreased number of intestinal pro-inflammatory Th-17 cells. However, no significant differences in fecal SCFA levels were observed in this context [10]. In parallel, studies in young healthy mice showed that KD decreases the abundance of pro-inflammatory bacteria, such as *Desulfovibrio* and *Turicibacter*, while promoting the production of beneficial microbes, such as *Akkermansia muciniphila* and *Lactobacillus*, which support production of SCFAs. These microbial shifts are accompanied by reduced blood glucose and increased levels of ketone bodies in circulation, consistent with sustained nutritional ketosis. Collectively, these alterations are associated with improved neurovascular function and reduced risk of neurodegeneration [11].

Compared to cognitively normal participants, individuals with mild cognitive impairment exhibit a relative increase in pro-inflammatory bacterial taxa and a reduction in bacteria associated with metabolic homeostasis at baseline, while fecal concentrations of acetate, propionate, and butyrate did not significantly differ [66]. Administration of a modified mediterranean KD reduces the abundance of harmful bacteria, such as *Bifidobacterium* and *Lachnobacterium*, while increasing beneficial microbes, including *Enterobacteriaceae*, *Akkermansia*, *Slackia*, *Christensenellaceae*, and *Erysipelotrichiaceae.* This shift enhances the production of SCFAs, particularly butyrate, which plays a critical role in maintaining gut integrity by reducing intestinal permeability and limiting the translocation of LPS into the bloodstream. These results suggest that KD can both support brain health and mitigate inflammation [66]*.*

Recent studies demonstrated that KD improved motor function and protected dopaminergic neurons in MPTP-induced mouse model of PD [68]. This effect was linked to increased SCFA-producing bacteria, such as *Blautia*, *Romboutsia,* and *Mycobacterium,* alongside a decrease in pro-inflammatory microbes, including *Desulfomicrobium* and *Alistipes.* These findings suggest that KD can modify gut microbiota to both alleviate neuroinflammation and enhance CNS resilience. Animal studies have consistently reported elevated levels of circulating ketone bodies during KD exposure, supporting a mechanistic association between sustained ketosis and microbiome remodeling [68, 69].

A well-balanced gut microbiome is essential for maintaining metabolic and neurological health, reducing oxidative stress, and providing potential neuroprotective effects. KD fosters a balanced gut environment, thereby mitigating factors associated with neurodegenerative diseases and enhancing overall brain health. However, the impact of KD on SCFA production appears to depend on dietary composition, disease context, and sustained nutritional ketosis.

## KD in specific neurodegenerative disorders

### AD

AD is the most prevalent neurodegenerative disorder and the most common cause of dementia worldwide; it is characterized by memory and cognitive dysfunction, and behavioral disturbances. It typically begins with subtle memory impairment and progresses to severe cognitive dysfunction and loss of independence over time [70]. Key pathological features of AD include the extracellular accumulation of Aβ plaques, derived from aberrant cleavage of the amyloid precursor protein (APP), and the formation of tau-containing neurofibrillary tangles. These abnormalities disrupt neuronal communication, leading to atrophy in areas such as the hippocampus and the neocortex, critical areas for learning and memory [70]. These pathological changes are accompanied by widespread inflammation, mitochondrial dysfunction, oxidative stress, and impaired cellular metabolism, which further contribute to neuronal damage [22, 71].

There is currently no cure for AD. Existing pharmacological therapies, such as acetylcholinesterase inhibitors and glutamate antagonists, aim to regulate neurotransmitter activity and provide modest symptomatic relief [72]. Impaired glucose metabolism is a hallmark of AD, which limits the ability of neurons to use glucose as their primary energy source, thereby exacerbating neuronal damage and cognitive decline [3]. Recent studies suggest that KD offers an alternative energy source through ketone bodies, which may compensate for this energy deficiency in the brain and stabilize neuronal activity [73]. Additionally, a clinical study found that high-glycemic diet is associated with increased cerebral amyloid burden in cognitively normal older adults, suggesting that diet is a potential modifiable behavior for cerebral amyloid accumulation. Therefore, dietary interventions like KD may reduce Aβ accumulation and lower the risk of AD [74].

Preclinical studies have demonstrated potential benefits of KDs (Table 1). Transgenic AD mouse models, including APP/PS1 and 5 × FAD mice, recapitulate key pathological features in AD patients, including progressive Aβ accumulation, plaque deposition, microglial activation, and synaptic dysfunction [16, 75]. A study in 2005 showed that the APP/V717I transgenic AD mouse model fed with KD for 43 days exhibited increased ketone body levels, reduced brain Aβ levels by 25%, and body weight loss, although the behavioral measures remained unchanged [16]. More recent studies in AD mouse models have indicated that KD improves cognitive function, alleviates neuroinflammation, reduces Aβ plaque deposition, and decreases microglial activation. These effects are associated with decreased levels of the microglial marker Iba1 and pro-inflammatory cytokines such as IL-1β and TNF-α, in some cases accompanied by reduced NF-κB activation, indicating modulation of inflammatory pathways associated with plaque progression and neuronal damage [22, 75, 76]. In 2024, a study in APP/PS1 transgenic mice revealed that KD and BHB supplementation restored long-term potentiation, a critical marker of synaptic plasticity linked to learning and memory. KD also increased BDNF levels in APP/PS1 female mice, reduced neuroinflammation by lowering microglial activation markers such as Iba-1 and CD11b [6], and enhanced ATP production [48]. These results support the hypothesis that KD can improve synaptic function and cellular energy metabolism in AD mouse models.

**Table 1:** Preclinical and clinical studies using ketogenic diet in Alzheimer's disease

Study	Animals/human subjects	Intervention	Duration	Outcome	References
Animal studies	APP/V717I transgenic AD mice (16 females)	KD	43 days	Significant increases in KB levels and a 25% reduction of brain Aβ levels; behavioral measures remained unchanged	[16]
	5 × FAD transgenic AD mice (only males)	KD	4 months	Improved cognitive function (spatial learning, spatial memory, and working memory), protected neurons and synapses, reduced Aβ deposition, decreased microglial activation and neuroinflammation	[75]
	APP/PS1 transgenic AD mice (only males)	KD	12 months	Improved cognitive function (learning and memory ability), reduced Aβ deposition and neuronal loss in the hippocampus of APP/PS1 mice	[76]
	APP/PS1 transgenic AD mice (only males)	KD	3 months	Enhanced cognitive function, reduced Aβ plaque deposition, and mitigated neuroinflammation by lowering microglial activation, proinflammatory cytokines (IL-1β, IL-6, TNF-α), and NF-κB levels while upregulating Nrf2	[22]
	APP/PS1 transgenic AD mice (both males and females were used)	KD and BHB	7–14 months	Restored long-term potentiation, increased BDNF levels in female mice, and reduced neuroinflammation by lowering microglial activation markers (Iba1 and CD11b)	[6]
	APP/PS1 transgenic AD mice (only males)	KD	3 months	Increased KB levels, enhanced ATP production, and attenuated microglial overactivation	[48]
Clinical studies	Twenty patients with mild-to-moderate AD (11 males, 9 females)	Single/Chronic administration of MCT-based ketogenic formula	12 weeks	The single dose of the MCT-based ketogenic formula showed increased ketone levels with no immediate cognitive benefits. Prolonged use suggested potential improvements in working memory, short-term memory, and processing speed in AD patients	[77]
	Fifty-two subjects with mild cognitive impairment (18 males, 21 females, and 13 dropped out)	Ketogenic drink containing MCT	6 months	Elevated KB levels and improvements in episodic memory, executive function, language, and processing speed	[78]

Studies summarized in Tables 1, 2, 3, 4, 5 were identified through a structured PubMed search using combinations of “ketogenic diet”, “ketone bodies”, or “β-hydroxybutyrate”, with specific neurodegenerative diseases, including Alzheimer’s disease, Parkinson’s disease, Huntington’s disease, amyotrophic lateral sclerosis, and multiple sclerosis. The search primarily focused on publications from the past 15 years. Only original preclinical and clinical studies published in peer-reviewed journals were included. Priority was given to studies reporting clearly defined ketogenic or ketone-based interventions and measurable mechanistic, metabolic, or clinical outcomes. Narrative reviews, opinion articles, and studies lacking clearly specified dietary protocols were not considered. AD, Alzheimer's disease; KD, Ketogenic diet; KB, Ketone bodies; Aβ, β-amyloid; IL, Interleukin; TNF-α, Necrosis factor-alpha; NF-κB, Nuclear factor kappa B; Nrf2, Nuclear factor erythroid 2-related factor 2; BHB, β-Hydroxybutyrate; BDNF, Brain-derived neurotrophic factor; CD11b, Cluster of Differentiation 11b; ATP, Adenosine triphosphate; MCT, Medium-chain triglyceride

Clinical studies have also highlighted the therapeutic potential of KD in humans. For example, Ota and colleagues demonstrated that a single dose of a medium-chain triglyceride (MCT)-based ketogenic formula increased plasma ketone levels, and continued administration was associated with cognitive improvement in AD patients [77]. Similarly, Fortier and colleagues reported that consumption of an MCT-based ketogenic drink for six months elevated ketone body levels and improved cognitive outcomes in subjects with mild cognitive impairment [78]. In 2021, Phillips et al*.* conducted the first randomized crossover trial investigating the effects of a modified KD for two 12-week treatment periods in AD patients. The patients achieved sustained ketosis and experienced improvements in daily function and quality of life without serious adverse effects [17].

In conclusion, KD presents a promising therapeutic approach for AD, although further research is needed to fully understand its potential and clinical applications.

### PD

PD is the second most common neurodegenerative disorder and the most prevalent neurodegenerative movement disorder, affecting approximately 1% of individuals over 60 years of age [79]. Clinically, PD is characterized by resting tremor, muscle rigidity, bradykinesia, and postural and gait difficulties. In addition to motor dysfunction, patients often exhibit non-motor symptoms, such as autonomic dysfunctions, cognitive changes, and sleep disturbances, which can precede motor symptoms and significantly impact quality of life [79].

The pathological hallmark of PD is the progressive loss of dopaminergic neurons in the substantia nigra, leading to striatal dopamine depletion. This is accompanied by abnormal α-synuclein deposition and the formation of Lewy bodies, which disrupt cellular function and contribute to neurodegeneration [80]. The etiology of PD is multifactorial and involves environmental and genetic factors, aging, and chemical exposure [81]. Despite the availability of dopaminergic treatments such as levodopa and dopamine agonists, current therapies are symptomatic and fail to halt or reverse disease progression. Moreover, prolonged use of levodopa is associated with complications such as dyskinesia and motor fluctuations, necessitating adjunctive strategies such as dopamine agonists and NMDA receptor antagonists to mitigate these side effects [80].

Dietary interventions, such as KD, have shown promise in addressing mitochondrial dysfunction, oxidative stress, and neuroinflammation, which are implicated in PD pathology [82] (Table 2). Pre-clinical studies demonstrated that KD and D-BHB protect neurons, improve mitochondrial function, and enhance ATP production particularly in models characterized by mitochondrial dysfunction and degeneration of dopaminergic neurons, which are hallmark pathological features of PD [39, 83].

**Table 2:** Preclinical and clinical studies using ketogenic diet in Parkinson's disease

Study	Animals/human subjects	Intervention	Duration	Outcome	References
Animal studies	MPTP-induced PD mouse model (only males)	D-BHB infusion	7 days	D-BHB attenuated the MPTP-induced dopaminergic neurodegeneration, preserved dopamine levels, and improved motor function by enhancing mitochondrial function and ATP production	[39]
	6-OHDA-induced rat model of PD (only males)	KD with MCT oils	25 days	KD increased serum ketone levels and enhanced motor function in PD rats	[84]
	MPTP-induced PD mouse model (only males)	KD	8 weeks	KD protected dopaminergic neurons and improved motor deficits in MPTP-induced PD mice by reducing inflammatory cytokine levels in the brain and plasma, and modulating gut microbiota	[68]
	MPTP-induced PD mouse model (only males)	MCT-KD	5 weeks	MCT-KD improved motor deficits and protected DA neurons in MPTP-induced PD mice by reducing oxidative stress through PI3K/Akt activation, reversing mitochondrial loss, and alleviating gut microbiota dysbiosis	[69]
Clinical studies	A pilot randomized controlled trial with 47 patients (38 completed the study)	KD	8 weeks	Improvement in the UPDRS Part 1 score (non-motor daily living experiences); improvements in motor and non-motor symptoms	[18]
	A randomized, controlled, parallel-group trial with 7 PD patients	Low-carbohydrate ketogenic regimen	8 weeks	Improvements in lexical access, memory, and body weight reduction; increased circulation of BHB; no effect on motor function	[85]
	A longitudinal pilot study in adults with PD (5 males and 2 females)	Low-carbohydrate ketogenic regimen	24 weeks	Positive effects on health biomarkers, cognition, mood, motor and non-motor symptoms, self-care, socialization, depression, and anxiety	[87]
	A pilot feasibility trial with 16 PD patients (15 completed the study)	KD supplemented with MCT oil	3 weeks	Improved energy and motor function; reduced fatigue; no significant changes in UPDRS between KD and the control group; significant reduction in non-motor severity scale scores	[86]

PD, Parkinson's Disease; MPTP, 1-methyl-4-phenyl-1,2,3,6-tetrahydropyridine; D-BHB, D-β-hydroxybutyrate; ATP, Adenosine triphosphate; KD, Ketogenic diet; 6-OHDA, 6-hydroxydopamine; MCT, Medium-chain triglyceride; DA, Dopaminergic; PI3K/Akt, Phosphoinositide 3-kinase/Protein kinase B; UPDRS, Unified Parkinson’s Disease Rating Scale; BHB, β-Hydroxybutyrate

In neurotoxin-induced mouse models of PD, KD reduces oxidative stress, rescues mitochondrial function, preserves tyrosine hydroxylase-positive neurons, and improves motor performance, reflecting improved dopaminergic neuronal survival and reduced ROS production [68, 69, 84]. Several studies have also reported that KD activates the antioxidant pathways, including Nrf2 signaling, supporting its role in modulating mitochondrial dysfunction and oxidative stress that contribute to dopaminergic neurodegeneration in PD models [69].

Clinical trials have corroborated these findings, with KD improving both motor and non-motor symptoms in PD patients [18, 85]. In 2024, Choi et al*.* reported that KD supplemented with MCT oil increased energy, reduced fatigue, and improved motor function in PD patients, despite no significant differences in the Unified Parkinson´s Disease Rating Scale score between the KD group and the control group [86]. Additionally, Tidman et al. demonstrated that adherence to a KD for 24 weeks significantly improved mobility, self-care, and several health-related biomarkers [87]. These findings highlight the potential of KD in managing PD, warranting further exploration of its long-term effects.

### HD

HD, initially described by George Huntington in 1872, is an autosomal-dominant neurodegenerative disorder characterized by involuntary movements, psychiatric symptoms, and cognitive deficits. It typically manifests in adulthood and follows an irreversible course, with symptoms progressively worsening over 15 to 20 years [88]. HD is caused by an abnormal expansion of the CAG trinucleotide in exon 1 of the Huntingtin (*HTT*) gene, which is located on chromosome 4. This mutation results in the production of a mutant protein with an elongated polyglutamine region, leading to the accumulation of protein aggregates in neurons, particularly in the basal ganglia and cerebral cortex. This accumulation causes widespread neuronal dysfunction and death [89].

Healthy individuals typically have 6–35 CAG repeats, whereas affected individuals have more than 36 CAG repeats. The severity of HD is linked to the number of CAG repeats, with larger expansions associated with earlier onset of symptoms [90]. The pathological mechanism of HD involves both a loss of function, due to impaired activity of the normal HTT protein, and a toxic gain of function from the mutant protein [91], resulting in oxidative stress, synaptic and mitochondrial dysfunction, impaired nuclear pore complex function, altered axonal trafficking, transcriptional dysregulation, and dysfunction of the ubiquitin–proteasome pathway [92].

While current treatments focus on symptom management, KD has emerged as a potential therapeutic approach that targets the underlying metabolic dysfunctions associated with HD (Table 3). Energy deficits in HD-affected neurons, caused by mitochondrial dysfunction, impaired glucose metabolism, and increased oxidative stress, are hallmark features of the disease. HD brains exhibit reduced activity of mitochondrial complexes II and III, leading to diminished ATP production and increased ROS, which exacerbate neuronal vulnerability [93]. KD provides an alternative energy source via ketone bodies, thereby enhancing mitochondrial function and compensating for impaired glucose metabolism [94].

**Table 3:** Preclinical and clinical studies using ketogenic diet in Huntington's disease

Study	Animals/human subjects	Intervention	Duration	Outcome	References
Animal studies	R6/2 1 J transgenic HD mice (males and females)	KD	10 weeks	No adverse effects on lifespan, working memory, or motor function; improvement in working memory in female mice; delayed onset of weight loss	[95]
	R6/2 transgenic HD mice (only males)	D-BHB infusion	10 weeks	Extended lifespan by ~ 30%; reduced the severity of motor deficits	[96]
	R6/2 transgenic HD mice	KD	7—10 weeks	Delayed weight loss; alleviated some behavioral symptoms; did not improve motor performance on the rotarod or prevent brain atrophy	[98]
Clinical study	A case study of a 41-year-old man with HD	Time-restricted KD	48 weeks	Improvement of motor symptoms (52%), daily living activities (28%), behavioral difficulties (50%–100%), mood-related quality of life (25%), and cUHDRS score (20%); no cognitive improvements	[99]

HD, Huntington's disease; KD, Ketogenic diet; D-BHB, D-β-hydroxybutyrate; cUHDRS, combined Unified Huntington’s Disease Rating Scale

Preclinical studies investigating KD in HD have primarily used transgenic mouse models, such as the R6/2 mice, which express mutant huntingtin and exhibit progressive motor deficits, weight loss, cognitive impairment, and mitochondrial dysfunction, mimicking central functional and metabolic aspects of HD progression [95, 96].

Ruskins and colleagues examined the effects of KD in the R6/2 1 J transgenic mouse model of HD over 10 weeks [95]. Compared with the standard diet, KD had no adverse effects on lifespan, working memory, or motor function. Notably, transgenic mice subjected to KD exhibited a delayed onset of weight loss. As weight loss is a hallmark of HD and maintenance of body weight has been associated with slower disease progression, this result suggests that KD may modulate metabolic alterations and energy imbalance in HD [97]. Female transgenic mice also showed improvements in working memory, suggesting cognitive benefits of KD in this mouse model [95].

Although the mechanisms remain unclear, the ability of KD to enhance mitochondrial function and increase energy production may contribute to these effects, as HD is also characterized by impaired mitochondrial activity and reduced cellular bioenergetic capacity [95]. Further corroborating evidence by Chen et al. demonstrated that KD delayed weight loss and alleviated some behavioral symptoms of R6/2 mice HD mice. However, KD failed to improve motor performance in the rotarod test or prevent brain atrophy, indicating that while KD may modulate metabolic aspects, it does not appear to fully prevent neurodegeneration in transgenic HD mice [98].

Given the limited therapeutic options for HD, Lim and colleagues investigated the neuroprotective properties of the ketone body D-BHB in R6/2 HD transgenic mice. They found that infusion of D-BHB extended lifespan by nearly 30% and ameliorated the severity of motor deficits, supporting the idea that improving the source of cellular energy may enhance neuronal survival and improve functional outcomes in HD models [96].

Early clinical evidence for KD in HD is promising but limited. A notable case study reported on a 41-year-old male patient with progressive, deteriorating HD, who adhered to a time-limited KD for 48 weeks. The patient experienced 52% improvement in motor symptoms, a 28% increase of daily living activities, and 20% improvement in cUHDRS (combined Unified Huntington’s Disease Rating Scale) score. Behavioral difficulties were improved by 50%–100%, and mood-related quality of life increased by 25% as assessed by standardized clinical scales, including the Unified Huntington´s Disease Rating Scale and patient-reported outcome measures [99]. These findings highlight the potential of KD as a therapeutic strategy in HD. Further research is needed to elucidate the underlying mechanisms and long-term effects.

### ALS

ALS is a fatal and rapidly progressive neurodegenerative disorder that primarily affects motor neurons in the brain and spinal cord, leading to muscle weakness, atrophy, and ultimately paralysis. ALS pathology involves degeneration of upper and lower motor neurons, misfolded protein accumulation (e.g., TDP-43), mitochondrial dysfunction, oxidative stress, neuroinflammation, and excitotoxicity [100]. The exact cause of ALS remains unclear, although genetic and environmental factors are known contributors. Mutations in superoxide dismutase 1 (*SOD1*), *C9orf72* (chromosome 9 open reading frame 72), and *TARDBP* (TAR DNA-binding protein 43) are associated with familial ALS, whereas the majority of ALS cases are sporadic [100, 101]. Clinically, ALS presents with progressive motor dysfunction, often starting with muscle weakness, fasciculations, and spasticity. Some patients experience cognitive or behavioral changes. In bulbar-onset ALS, speech, swallowing, and respiratory function may also be impaired [100].

Despite significant research efforts, ALS remains incurable. Approved treatments such as riluzole and edaravone offer only modest benefits. Riluzole slightly extends survival by reducing glutamate toxicity, whereas edaravone may slow functional decline in selective early-stage patients. These limited effects highlight the need for more effective therapeutic approaches [100]. Given the complex pathology of ALS, KD has been proposed as a potential therapeutic approach (Table 4). Emerging evidence suggests its ability to modulate mitochondrial function, reduce neuroinflammation, and improve energy metabolism [40, 102].

**Table 4:** Preclinical and clinical studies using ketogenic diet in amyotrophic lateral sclerosis

Study	Animals/human subjects	Intervention	Duration	Outcome	References
Animal studies	SOD1-G93A transgenic ALS mice (11 males)	KD	Till the study endpoint	Blood ketone levels were 3.5 times higher in KD-fed animals compared to controls; preserved motor function and motor neurons in the lumbar spinal cord; slowed weight loss	[40]
	SOD1-G93A transgenic ALS mice (males)	Caprylic triglyceride	Till the study endpoint	Blood ketone levels were 2.5 times higher than in controls; significantly higher numbers of motor neurons in the lumbar spinal cord; improvements in motor function and mitochondrial oxygen consumption; no significant differences in weight, food consumption, and survival outcomes	[102]
Clinical study	A case study of a 64-year-old man with bulbar-onset ALS	Time-restricted KD	18 months	ALS-related function improved by 7%, forced expiratory volume improved by 17%, forced vital capacity improved by 13%, quality of life improved by 19%; depression and stress levels normalized; weight loss was minimized; swallowing and cognitive function remained stable	[103]

ALS, Amyotrophic lateral sclerosis; KD, Ketogenic diet

Preclinical studies have evaluated KD primarily in *SOD1* transgenic mouse models, which exhibit progressive motor neuron degeneration, muscle weakness, and mitochondrial dysfunction, thereby recapitulating key features of disease progression [40, 102]. Zhao et al. demonstrated that KD preserved motor function and slowed weight loss in SOD1-G93A transgenic ALS mice [40]. Additionally, the KD-fed mice showed elevated blood ketone levels and preserved motor neurons in the lumbar spinal cord, indicating improved motor neuron survival [40]. Importantly, by isolating spinal cord mitochondria from ALS transgenic mice, researchers found that D-BHB supplementation enhanced ATP production in mitochondria, supporting the idea that ketone bodies may improve mitochondrial function and cellular bioenergetics in affected motor neurons [40]. Another study showed that the administration of caprylic triglyceride, a precursor of ketone bodies, improved motor function and mitochondrial oxygen consumption rates in G93A ALS transgenic mice, indicating that KD may enhance mitochondrial respiration and functional performance in ALS. However, the survival was not significantly improved [102].

A case study of a 64-year-old male with a 21-month history of progressive bulbar-onset ALS [103] highlighted the potential benefits of KD. After 18 months of KD, the patient showed a 7% improvement in ALS-related function, a 17% increase in forced expiratory volume, and a 13% improvement in forced vital capacity. Depression and stress levels were normalized, and quality of life improved by 19%. Notably, the patient maintained stable swallowing and cognitive function and minimal weight loss. He reported better energy levels, improved sleep, and reduced chronic musculoskeletal pain without adverse effects [103]. The 64-year-old patient remained functionally independent over 45 months since symptom onset and continued to adhere to the KD. This case represents the first documented instance of ALS management, providing encouraging preliminary evidence for KD as a potential therapeutic approach [103].

### MS

MS is a chronic autoimmune disorder characterized by inflammatory demyelination, axonal injury, and progressive neurodegeneration within the CNS, affecting approximately 2.8 million people worldwide. It typically manifests in young adults, between 20 and 40 years of age, with females being two times more likely to have MS [104, 105].

Clinically, MS is characterized by recurrent episodes of neurological dysfunction, including optic neuritis, sensory alterations, motor weakness, gait disturbances, and cognitive impairment. The most frequent disease course, relapsing–remitting MS, involves acute inflammatory relapses followed by periods of partial recovery. Over time, a substantial proportion of patients develop a progressive phase, typically 10 to 15 years after disease onset, characterized by gradual accumulation of disability and ongoing neurodegeneration that becomes less directly associated with the acute inflammatory activity [104].

Pathologically, MS is defined by focal demyelinating lesions associated with inflammatory infiltrates containing T cells, B cells, and activated microglia [106, 107]. Axonal damage and neuronal loss occur early in the disease progression and contribute significantly to long-term disability [108]. In addition to immune-mediated myelin damage, mitochondrial dysfunction and impaired energy metabolism have emerged as central contributors to axonal degeneration [109]. Demyelinated axons exhibit increased energy demand, impaired mitochondrial transport, and reduced ATP production, which increase their vulnerability to metabolic stress [110, 111].

Several disease-modifying therapies are currently approved for MS, primarily targeting the inflammatory mechanisms. Interferon-β, which shifts the cytokine balance toward an anti-inflammatory profile, is one of the first treatments shown to reduce relapse rates in relapsing–remitting MS [112]. Monoclonal antibodies that limit immune cell trafficking, such as natalizumab, reduce the relapse frequency and slows the disability progression [113]. B-cell-depleting therapies, including ocrelizumab, reduce disease activity in MS, further confirmed the central role of B cells in MS pathogenesis [114]. Despite these advances, current therapies mainly modulate immune responses, but do not fully prevent progression of neurodegeneration.

Given the prominent role of inflammation, oxidative stress, and mitochondrial dysfunction in MS pathophysiology, metabolic interventions aimed at enhancing mitochondrial function and providing an alternative energy source, such as the KD, have been explored as potential adjunctive strategies to slow disease progression and improve clinical outcomes (Table 5).

**Table 5:** Preclinical and clinical studies using ketogenic diet in multiple sclerosis

Study	Animals/human subjects	Intervention	Duration	Outcome	References
Animal studies	EAE mouse model	KD	Till the study endpoint	Decreased disease severity; improved motor performance; decreased CNS inflammatory cytokines, chemokines, and ROS levels	[115]
	Cuprizone-induced demyelination in mice (only males)	KD	35 days	Significantly less weight loss; serum levels of BHB increased in KD animals; attenuated demyelination; reduced the expression of TNF-α and IL-1β; decreased expression of NLRP3	[117]
	EAE mouse model (only females)	KD	24 days	Improved motor outcomes; inhibited demyelination; improved pathological lesions; suppressed microglial activation; inhibited NLRP3 and NF-κB activation; reduced pro-inflammatory cytokines	[116]
Clinical studies	Randomized controlled trial, with 11 MS patients in KD	Adapted KD	6 months	Decreased *COX-2* and *ALOX5* gene expression; modulation of inflammatory signaling pathways	[120]
	Randomized controlled trial with 17 MS patients in KD	Adapted KD	6 months	Reduced serum neurofilament light chain levels, suggesting modulation of neuroaxonal injury biomarker	[121]
	Phase 2 trial with 64 relapsing–remitting MS patients	Modified Atkins KD	6 months	Improved fatigue, depression, and quality of life; reduced levels of pro-inflammatory adipokine (leptin) and increased levels of anti-inflammatory adipokine (adiponectin)	[118]
	Randomized controlled trial with 36 relapsing–remitting MS patients in KD	KD	18 months	Improved fatigue, quality of life measures, and disability scores; reduced levels of leptin and increased levels of adiponectin with 9 months of treatment	[119]

MS, Multiple sclerosis; EAE, autoimmune encephalomyelitis; KD, Ketogenic diet; CNS, Central nervous system; ROS, reactive oxygen species; BHB, β-Hydroxybutyrate; TNF-α, Necrosis factor, alpha; IL, Interleukin; NLRP3, Nucleotide-binding oligomerization domain-, leucine-rich repeat, and pyrin domain-containing protein 3; NF-κB, Nuclear factor kappa B; COX-2, Cyclooxygenase-2; ALOX5, 5-lipoxygenase

Preclinical studies in experimental autoimmune encephalomyelitis (EAE), a widely used animal model of MS, showed that KD reduced disease severity, improved motor performance, decreased production of inflammatory cytokines (IL-1β, IL-6, and TNF-α) and ROS [115]. Additionally, mechanistic studies further demonstrated that KD modulates inflammatory signaling pathways in EAE models, including reducing NF-κB activation and decreasing NLRP3 inflammasome activity, alongside improvements in motor outcomes [116]. In toxin-induced demyelination models, such as the cuprizone model, KD attenuated demyelination, as demonstrated by histological assessment and preservation of myelin basic protein. KD also prevented microglial activation, which may correlate with decreased expression of NLRP3 and reduced production of pro-inflammatory cytokines (TNF-α and IL-1β), supporting a potential role in mitigating inflammatory and metabolic stress during demyelination [117].

Clinical investigations remain limited, but they support the feasibility and the translational relevance of KD in MS. In a 6-month Phase II study of a modified Atkins KD in patients with relapsing–remitting MS, the intervention was well tolerated, improved fatigue, depression, and quality of life, reduced the levels of pro-inflammatory adipokines, such as leptin, and increased the level of adiponectin, an anti-inflammatory adipokine [118]. A randomized controlled trial with 18-month follow-up in patients with relapsing–remitting MS reported sustained tolerability and improvements in fatigue, quality of life, and disability scores in the KD group [119]. These studies suggest that metabolic modulation may influence the symptom burden in inflammatory diseases.

Beyond clinical outcomes, the effects on biomarkers were also evaluated. The levels of serum neurofilament light chain, a marker of neuroaxonal injury, were significantly reduced by 6-month adapted KD in MS patients, suggesting that metabolic modulation may influence neurodegeneration markers. In addition, KD was associated with reduced expression of inflammatory genes, including *COX-2* and *ALOX5* (5-lipoxygenase), further supporting a link between ketosis and modulation of immune-related signaling pathways in MS [120, 121].

Collectively, these findings suggest that KD may modulate inflammatory signaling and metabolic stress in MS. Larger controlled studies are needed to evaluate the long-term efficacy and potential impact of KD on disease progression.

## Challenges for clinical application

Although growing evidence supports the therapeutic potential of KD in neurodegenerative disorders, its practical application in clinical settings remains challenging. Key issues include patient adherence and compliance with the strict dietary regimen, safety concerns associated with long-term use, and questions regarding the diet’s sustainability over extended periods [26, 122]. To optimize KD as a therapeutic option, further studies are needed to evaluate its long-term efficacy, potential side effects, and strategies to enhance patient compliance. Addressing these gaps is crucial for translating promising preclinical findings into feasible and effective clinical interventions for patients with neurodegenerative disorders.

### Patient adherence and compliance

Patient adherence is a critical factor of the effectiveness of KD. The strict and complex dietary regimen of KD often leads to refusal to participate or withdrawal from clinical studies [86]. Additional factors contributing to non-compliance include side effects, psychosocial challenges, and limitations in social activities [123]. Efforts to improve adherence include educational tools, such as multimedia resources and weekly instructional videos [18]. Alternative dietary approaches, such as the Modified Atkins Diet, have also been explored to reduce restrictions while maintaining the therapeutic efficacy. Unlike KD, this diet does not restrict protein, fluids, or calorie intake and limits carbohydrates to 20 g per day, making it more palatable and easier to follow [26].

Challenges related to meal preparation and dietary adherence were evident in a randomized feasibility trial of KD in PD patients [86]. Phillips et al*.* addressed these challenges by creating tailored meal plans, shopping lists, and affordable menus that preserved palatability while monitoring daily blood glucose and ketone levels [17, 18]. Similarly, Tidman et al. implemented weekly communication during a 12-week intervention to provide guidance, address concerns, and adjust meal plans based on feedback. However, patients still reported challenges such as higher diet costs, lack of family support, difficulties during holiday meals and social events, and the exclusion of favorite foods like fruit [123].

In addition, most studies still focus on the short-term applications of KD, with limited exploration of long-term adherence strategies, particularly in neurodegenerative patients. This underscores the need for further research on behavioral and social interventions to improve compliance in both clinical and everyday contexts.

### Long-term effects of KD

The long-term safety profile of KD remains a concern due to its unconventional macronutrient distribution, characterized by high fat and low carbohydrate intake. Common adverse effects reported by epilepsy patients include weight loss, gastrointestinal issues, and transient hyperlipidemia [122, 124]. Short-term side effects, often referred to as “keto flu”, typically occur during the initial weeks of the diet. These include headache, fatigue, nausea, dizziness, gastrointestinal discomfort, hypoglycemia, dehydration, and decreased energy, which generally subside as the body adapts to ketosis [125]. Gastrointestinal issues, such as constipation, diarrhea, nausea, vomiting, and occasionally pancreatitis, are among the most common side effects. These effects are largely attributed to the diet’s low fiber content and can often be mitigated by increasing dietary fiber, sodium, and fluid intake, consuming smaller meals, or using enemas or polyethylene glycol [126, 127]. The restriction of carbohydrate-rich foods in KD can lead to deficiencies in vitamins and minerals, including thiamin, folate, vitamin A, vitamin E, vitamin B6, calcium, magnesium, iron, and potassium [128]. Supplementation with the recommended daily allowance of multivitamins and minerals can prevent such deficiencies [17].

Long-term adverse effects, although less common, include elevated cardiovascular risks due to poor cholesterol profiles (increased triglycerides, cholesterol, and low-density lipoprotein cholesterol), decreased bone mineral density, nephrolithiasis, anemia, neuropathy, hepatic dysfunction, and atherosclerosis [124, 129].

For neurodegenerative diseases, especially in older patients, KD must be tailored to address underlying health conditions and nutritional risks. In this population, unintended weight loss, inadequate protein intake, or micronutrient deficiencies may occur if dietary supervision is insufficient. These patients often face challenges such as chewing and swallowing difficulties, altered taste and smell, apraxia, disordered eating behaviors, and motor-related issues that hinder meal preparation and consumption [88].

A randomized controlled trial in AD patients reported no significant adverse effects, with irritability being the most common complaint [17]. In PD studies, adverse effects include worsening of symptoms such as tremor and stiffness along with fatigue, headache, constipation, increased irritability, and heightened sensations of hunger or thirst [18, 86, 123]. Adjustments in the dietary regimen, including addition of electrolytes and increased water intake, help alleviate some of these issues [123]. In HD patients, the side effects reported include mild weight loss, decreased bowel movement frequency, and increased thirst, none of which require medical intervention [99].

While KD shows promise as a therapeutic approach for treating neurodegenerative diseases, further studies are needed to evaluate its long-term safety and efficacy. Current clinical research in humans often relies on small sample sizes, short intervention periods, and self-reported data, leading to variability in outcomes. Future studies should aim to refine the diet to reduce side effects, improve adherence, and establish robust clinical evidence, paving the way for tailored KD-based therapies.

## Conclusion

KD has emerged as a metabolically oriented strategy with potential preventive and therapeutic relevance in neurodegenerative diseases. Its ability to influence critical biological pathways, including mitochondrial function, oxidative stress, neuroinflammatory processes, and cellular bioenergetics, positions KD as an important nonpharmacological approach capable of addressing metabolic disturbances shared across disorders such as AD, PD, HD, ALS, and MS.

While preclinical studies have demonstrated encouraging results, significant gaps remain in understanding long-term effects, safety, and practicality of KD in clinical settings. Importantly, KD may serve as a complementary metabolic intervention that supports disease-specific treatments by enhancing metabolic resilience and contributing to symptom management. Additionally, adherence to such a restrictive dietary regimen poses a major challenge, highlighting the need for practical strategies to make KD more accessible and sustainable for patients.

Future research should prioritize personalizing KD through advanced genetic and metabolic profiling, enabling predictions about which patients are most likely to benefit. Emerging technologies, such as wearable devices for biomarker discovery, can enhance monitoring, adherence, and outcome tracking. Combining KD with other dietary interventions, such as the Mediterranean diet, may also amplify therapeutic effects by targeting complementary pathways involved in disease progression. Importantly, further studies are needed to determine whether early implementation of KD may delay disease onset or attenuate initial metabolic and inflammatory disturbances in at-risk populations, thereby clarifying its preventive potential alongside its therapeutic application.

In conclusion, while KD is not a definitive cure, its capacity to modulate key disease mechanisms offers promise for improving outcomes in individuals with neurodegenerative diseases. Refining and expanding the application of KD is critical to establish it as an effective tool for managing these debilitating conditions.

## Data Availability

Not applicable.

## Abbreviations

AcAc: Acetoacetate
acetyl-CoA: Acetyl coenzyme A
AD: Alzheimer’s disease
ALS: Amyotrophic lateral sclerosis
AMPK: AMP-activated protein kinase
APP: Amyloid precursor protein
ATP: Adenosine triphosphate
Aβ: β-Amyloid
Bcl-2: B-cell lymphoma 2
BDNF: Brain-derived neurotrophic factor
BHB: β-Hydroxybutyrate
C9orf72: Chromosome 9 open reading frame 72
COX-2: Cyclooxygenase-2
D-BHB: D-β-Hydroxybutyrate
EAE: Autoimmune encephalomyelitis
HCA2: Hydroxycarboxylic acid receptor 2
HD: Huntington’s disease
HMG-CoA: Mitochondrial 3-hydroxy-3-methylglutaryl-CoA
HTT: Huntingtin
ILs: Interleukins
KD: Ketogenic diet
LPS: Lipopolysaccharides
MCT: Medium-chain triglyceride
MS: Multiple sclerosis
mTORC1: Mammalian target of rapamycin complex 1
NADH: Nicotinamide adenine dinucleotide hydrogen
NF-κB: Nuclear factor kappa B
NLRP3: Nucleotide-binding oligomerization domain-, leucine-rich repeat- and pyrin domain-containing protein 3
NMDA: *N*-Methyl-D-aspartate
Nrf2: Nuclear factor erythroid 2-related factor 2
PD: Parkinson’s disease
PGC-1α: Peroxisome proliferator-activated receptor γ coactivator 1-α
ROS: Reactive oxygen species
SCFAs: Short-chain fatty acids
SIRT1: Sirtuin-1
SOD1: Superoxide dismutase 1
TCA: Tricarboxylic acid
TNF-α: Necrosis factor-alpha
